# Hyperspectral Analysis for Discriminating Herbicide Site of Action: A Novel Approach for Accelerating Herbicide Research

**DOI:** 10.3390/s23239300

**Published:** 2023-11-21

**Authors:** Zhongzhong Niu, Tanzeel Rehman, Julie Young, William G. Johnson, Takayuki Yokoo, Bryan Young, Jian Jin

**Affiliations:** 1 Department of Agricultural and Biological Engineering, Purdue University, West Lafayette, IN 47907, USA; niu38@purdue.edu (Z.N.); yokoot@sc.sumitomo-chem.co.jp (T.Y.); 2 Department of Biosystems Engineering, Auburn University, Auburn, AL 36849, USA; tur0001@auburn.edu; 3 Department of Botany and Plant Pathology, Purdue University, West Lafayette, IN 47907, USA; young294@purdue.edu (J.Y.); wgj@purdue.edu (W.G.J.); bryanyoung@purdue.edu (B.Y.); 4 Health and Crop Sciences Research Laboratory, Sumitomo Chemical Co., Ltd., Takarazuka 665-8555, Hyogo, Japan

**Keywords:** hyperspectral imaging, herbicide, site of action, machine learning, support vector machines (SVM), partial least squares discriminant analysis (PLS-DA)

## Abstract

In agricultural weed management, herbicides are indispensable, yet innovation in their modes of action (MOA)—the general mechanisms affecting plant processes—has slowed. A finer classification within MOA is the site of action (SOA), the specific biochemical pathway in plants targeted by herbicides. The primary objectives of this study were to evaluate the efficacy of hyperspectral imaging in the early detection of herbicide stress and to assess its potential in accelerating the herbicide development process by identifying unique herbicide sites of action (SOA). Employing a novel SOA classification method, eight herbicides with unique SOAs were examined via an automated, high-throughput imaging system equipped with a conveyor-based plant transportation at Purdue University. This is one of the earliest trials to test hyperspectral imaging on a large number of herbicides, and the study aimed to explore the earliest herbicide stress detection/classification date and accelerate the speed of herbicide development. The final models, trained on a dataset with nine treatments with 320 samples in two rounds, achieved an overall accuracy of 81.5% 1 day after treatment. With the high-precision models and rapid screening of numerous compounds in only 7 days, the study results suggest that hyperspectral technology combined with machine learning can contribute to the discovery of new herbicide MOA and help address the challenges associated with herbicide resistance. Although no public research to date has used hyperspectral technology to classify herbicide SOA, the successful evaluation of herbicide damage to crops provides hope to accelerate the progress of herbicide development.

## 1. Introduction

Herbicides have played an important role in weed control for about 70 years [1]. Today, herbicides account for about 60% of the pesticides used worldwide, and most large-scale farming systems rely largely on synthetic herbicides for weed control [2,3,4,5]. The vital importance of herbicides for weed control was reflected in extensive herbicide discovery efforts for several decades prior to the 1990s. However, the widespread adoption of herbicide-tolerant crops and the corresponding increase in the use of the broad spectrum and cost-efficient herbicide glyphosate in the mid-1990s negatively impacted herbicide discovery efforts. In the last 30 years, not a single herbicide with a new mode of action (MOA) has been launched [6,7]. Discovery efforts were suppressed not only by the shift in focus to crops tolerant to existing herbicides but also by the tremendous cost associated with registering a new active ingredient. Traditional methods for identifying herbicide MOA often involve biochemical assays and molecular docking studies [8,9]. These conventional approaches are not only time-consuming but may also require significant financial investment [10]. Bayer has announced a 10-year strategic initiative to develop novel herbicides aimed at combating weed resistance to glyphosate, with a projected investment of $5.6 billion in 2020 [11].

Despite these significant financial and regulatory concerns, research to discover new herbicide MOA is critical for the agricultural industry due to the widespread problem of herbicide-resistant weeds [12,13]. Overuse of glyphosate in the control of broadleaf weeds led to weed resistance development [3]. Worldwide, weeds have developed resistance to 21 herbicide site-of-action groups [14]. New technologies for herbicide SOA discovery and analysis with lower costs are urgently needed to accelerate research and slow the damaging progression of herbicide resistance. 

Hyperspectral technology offers a potential solution for the discovery of new herbicides SOA with its advantages of high throughput and non-invasive assessment features. Hyperspectral imaging combined with machine learning has been used to assess the damage caused by herbicides to crops in different doses. Ting Zhang (2021) selected plant physiological reflectance index (PRI) and normalized difference vegetation index (NDVI) from hyperspectral images (HSI) using machine learning methods, including support vector machine (SVM) to classify the damage of maize by different doses of glyphosate [15]. Huangjian Chu (2022) used HSI with neural networks to classify three different types of herbicide damage on wheat. They found spectral reflectance exhibited obvious differences at 518–531 nm, 637–675 nm, and the red edge [16]. Zhongzhong Niu (2022) developed a PLS-DA method that can distinguish soybean damage caused by off-target dicamba and 2,4-D using spectral and texture features extracted from HSI [17]. Although there is no public research to date that has used hyperspectral technology to classify multiple herbicides SOA on weed, the successful evaluation of herbicide damage to crops provides hope. 

The aim of this study is to advance the classification of herbicide Sites of Action (SOA) by deploying hyperspectral imaging technology. In particular, the classification will focus on eight distinct SOAs, with the identification of spectral signatures being paramount. The outcome expected from this methodology is a refined understanding of SOA characteristics, which will assist in the rapid identification of new MOAs. This research is poised to provide significant advancements in herbicide development, offering agricultural scientists and agrochemical companies a potent tool to counteract the challenge of herbicide-resistant weeds and promote sustainable agricultural methods.

## 2. Materials and Methods

### 2.1. High-Resolution Hyperspectral Imaging Acquisition System

**Figure 1:**
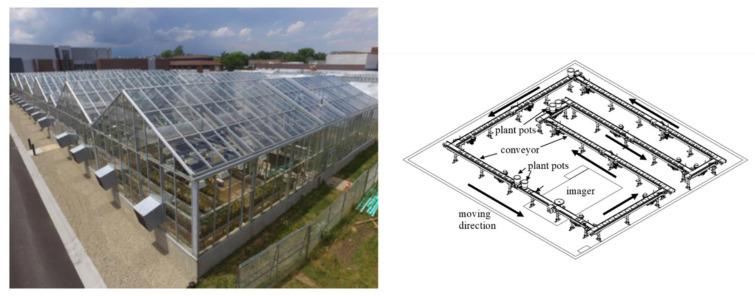
Image of Purdue Lily Greenhouse with automated, high-throughput imaging system with a belt conveyor-based plant transportation system (**left**) and its layout (**right**), adapted from [8].

The experiment was conducted in the imaging greenhouse at Purdue University Lily Department (latitude 40.4259° N, longitude 86.9081° W). This imaging system with automatic transportation conveyors can effectively remove the greenhouse microclimate heterogeneity to keep the collected HIS data in high quality [18]. The image of this greenhouse facility is shown in Figure 1.

An indoor high-resolution hyperspectral imaging system was constructed specifically for this study. The system utilized a line-scanning hyperspectral camera (MSV-500, Middleton Spectral Vision Co., Middleton, WI, USA) with a spatial resolution of 1000 pixels per line and a spectral range of 380–1030 nm. Table 1 provides additional details about the camera specifications. The imaging system employed an illumination setup with two halogen light sources (MRC-920-029, Middleton Spectral Vision Co., Middleton, WI, USA) to provide consistent lighting for the samples. The system was capable of simultaneously recording two grass samples within a time span of approximately one minute per sample. The camera was positioned horizontally to capture a side view of the barnyard grass plant, which was placed on a white background to minimize the impact of surrounding environmental factors on the hyperspectral images. Overall, the imaging system was carefully designed and constructed to provide high-quality hyperspectral images of the barnyard grass samples in a controlled indoor environment. The use of consistent illumination and a white background helped minimize potential confounding factors in the acquired hyperspectral data.

### 2.2. Experiment Design

**Figure 2:**
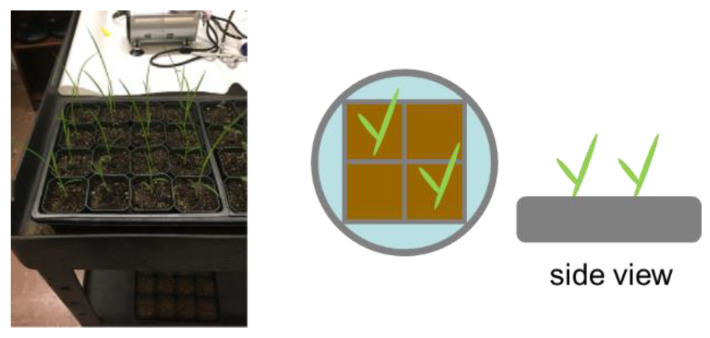
Barnyard grass was used for the experiment. The top and side views of the layout are illustrated.

Barnyard grass (*Echinochloa crus-galli*) was selected as the subject in this experiment. The plants were grown in 5 cm square pots filled with a 2:1 (*v*/*v*) potting mix/sand mixture (Figure 2), and herbicide treatments were applied 10 days after sowing when the barnyard grass had two leaves. The selected herbicide doses (Table 2) were applied to 16 samples of each herbicide treatment using an atomizer calibrated to deliver the specific dose. The herbicides were chosen to represent four different herbicide MOA groups (Table 3), each with at least two unique SOAs. The study aimed to investigate the efficacy of the different herbicides on barnyard grass control.

For continuously monitoring the stress of herbicide developed, hyperspectral image recording was conducted starting from the second hour after treatment (1 DAT) and continuing daily until the seventh day after treatment (7 DAT). To ensure the reliability and robustness of the results, the experiment was conducted in two runs, with the second run serving as a repeat of the first run with the same number of treatments and samples, analyzing a cumulative total of 320 samples. 

### 2.3. Image Processing and Mean Spectrum Extraction

The acquired raw hyperspectral images were calibrated using a flat polyvinyl chloride (PVC) board to effectively reduce the non-uniformity of the light source across all wavelengths, as described in [19]. Five plant samples severely damaged by herbicide on 2 DAT were considered outliers of the dataset. The calibration process is presented in the following equation:

$$R_{c}=\frac{{R_{raw}-R}_{dark}}{{R_{white}-R}_{dark}}$$

The calibrated images were then further processed to segment out the plant pixels via a red edge segmentation algorithm proposed by Zhang et al. [20]. Using the wavelengths 680–732 nm as the characteristic vegetation areas of the spectrum, it is possible to segment the barnyard grass tissue from the background. Below is the algorithm:

(1) $$lin=transpose\left(-20:20\right)$$

(2) $$con=\;lin*transpose(squeeze\left(img\left(:,:,I_{680}:I_{732}\right)\right))/(lin*lin)$$

(3) plt=con>threshold

A one-dimensional vector of sequential integers ranging from −20 to 20 (represented as *lin*) was applied to enlarge the difference between plants and background in the NIR region (Equation (2)). A threshold of 7 was determined to yield optimal segmentation outcomes in Equation (3). An average was calculated over all the plant pixels to extract the mean spectrum. Due to a better signal-to-noise (SNR) ratio, only the bands in the 460–975 nm range were kept from the mean spectral data. 

**Figure 3:**
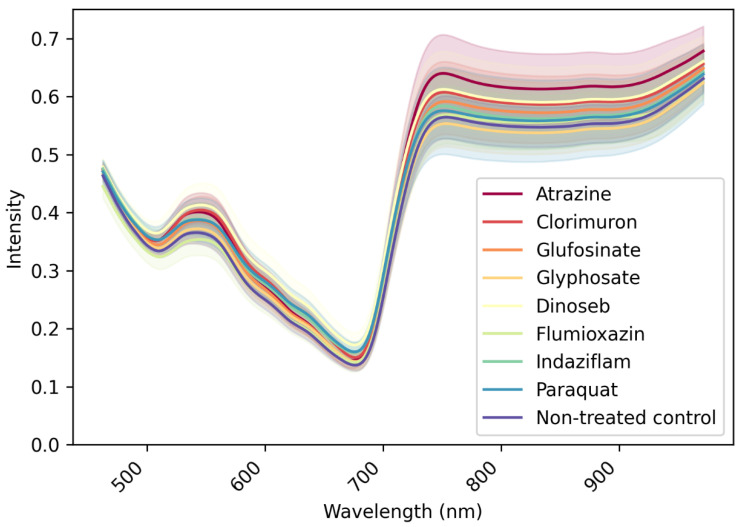
Mean spectral response for each treatment on 1 DAT of round 1 experiment, with shaded regions indicating the 95% confidence intervals. The *x*-axis represents wavelength (in nanometers), and the *y*-axis denotes reflectance or absorbance values.

The spectral data was pre-processed with the Savitzky–Golay smoothing filter of order 1 with a window size of 5. The Savitzky–Golay smoothing filter is effective on the spectrum for noise reduction [21]. Image processing and analysis were carried out using MATLAB^®^ 2020a (MathWorks Inc., Natick, MA, USA) image processing toolbox. An example of the extracted average reflectance spectral data is illustrated in Figure 3. 

### 2.4. Pairwised T-Test for NDVI

The Normalized Difference Vegetation Index (NDVI) is a widely utilized metric for assessing vegetation health and detecting stress signals [15,22]. In this study, NDVI values of each sample were calculated using data from one day after treatment. To statistically analyze the differences between various treatments, a pairwise *t*-test was applied to all the pairs of herbicide treatment and control groups using Python. A heatmap with all the *p* values from the *t*-test was generated to visualize the result.

### 2.5. Feature Selection Using Random Forest and One-vs-All Approach

The Random Forest algorithm, an ensemble learning method, was utilized for feature selection, determining the importance of each band (feature) in differentiating between treatments [23]. Each treatment was considered as a separate class in a one-vs-all approach, which allowed for the evaluation of band importance for each treatment individually. The Random Forest parameters were set as follows: n_estimators = 100, denoting the number of decision trees in the forest to ensure stability in the importance scores, and random_state = 42, a seed used by the random number generator to ensure reproducibility of results. 

### 2.6. Machine Learning Method

Two algorithms, partial least squares discriminant analysis (PLS-DA) and support vector machines (SVM), were compared for the classification of herbicides with different SOA. Different pre-processing strategies, including transformation, scaling, and scatter corrections, were optimized for PLS-DA and SVM models [10]. The final pre-processing combination steps were applied to the average spectrum for both PLS-DA and SVM. Then, they were ordered as log(1/Reflectance) followed by mean scatter correction (MSC) and concluded with mean centering (MC). These pre-processing steps were conducted utilizing the PLS_Toolbox from Eigenvector Research, Inc., with specific parameters and further details referenced in the official documentation [24]. Leave-one-out CV was used for developing all the models reported in this study. The PLS-DA models were trained using as many as three latent variables (LVs), with the best model being the one with the smallest cross-validated root mean square error (RMSEcv). This study used linear and radial basis function (RBF) kernels for the SVM models [25,26,27]. An exhaustive grid search was used for optimizing the regularization parameter (C) and RBF kernel coefficient (γ) in a range of 1 × 10^−3^ to 100, 1 × 10^−6^ to 1 × 10^−1^. The SVM model with the best combination of kernels, C, and/or γ parameters was selected based on the smallest RMSEcv.

To classify multiple SOAs, we tested a one-vs-one (OVO) modeling paradigm for both PLS-DA and SVM. In OVO, the SOA data were divided into multiple binary classification problems. A binary classifier was trained per pair of SOA classes. To predict the final outcome class from different binary classifiers, we used an ensemble-based soft voting mechanism. For each binary classifier, we extracted the cross-validated prediction probabilities of each class and computed the average probability of a sample belonging to a specific class. The OVO, assisted with soft voting, assigned the final label to a class having maximum average probability.

To compare the performance of OVO models for PLS-DA and SVM, round 1 data from 1–4 DAT were used to train the models. For each PLS-DA model’s validation, leave-one-out method was used. The machine learning methods with higher performance were chosen to train a more stable model with two rounds of data 1–7 DAT combined. Two rounds of datasets are used as training datasets to build the final herbicide SOA model. There are 32 samples for each herbicide treatment and 64 for the control group. A day-to-day validation method was used to validate the models with the highest performance among all the seven days. The term “day-to-day” validation refers to a cross-validation technique where the model is trained on spectral data from one day and subsequently tested on data from a different day. This approach is designed to assess the model’s robustness to daily variations in the data.

Overall Accuracy (OA) and error rate were used to examine the performance of models. They were calculated using the Equations (4) and (5):

(4) $$OA=\frac{Numbr\;of\;Correct\;Predictions}{Total\;Number\;of\;Predictions}$$

(5) Error Rate=1−OA

## 3. Results

### 3.1. NDVI T-Test Results and Featued Bands for SOA Classsification

**Figure 4:**
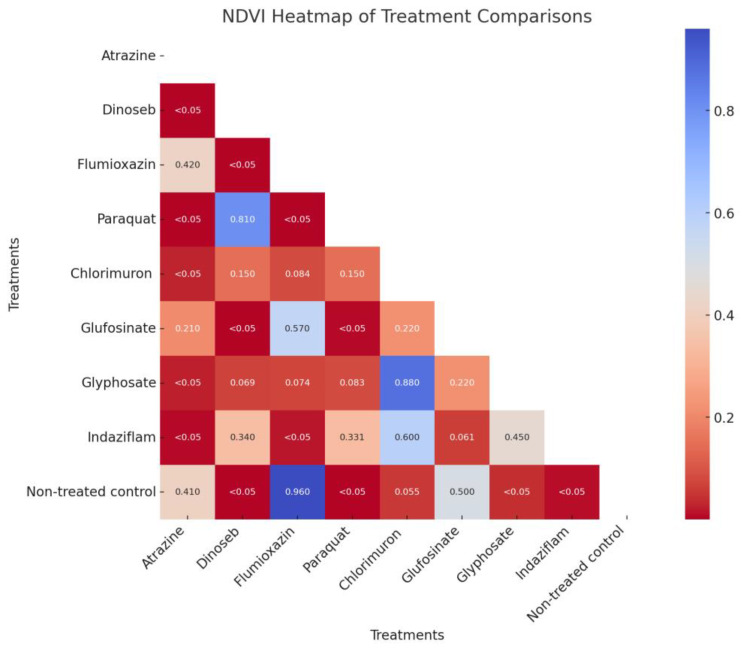
Heatmaps of *p*-values from pairwise *t*-tests of NDVI between treatments. The colors in the heatmap transition from blue to red, with blue representing lower NDVI differences and red signifying higher NDVI differences.

The heatmap analysis (Figure 4) of pairwise comparisons of NDVI values across various herbicide treatments reveals distinct patterns. Approximately half of the treatment pairs exhibit significant differences, with *p*-values less than 0.05. Atrazine (PS II inhibition) shows significant differences with several treatments, whereas Dinoseb (Uncoupler) displays significant differences with the control group but has a high *p*-value (0.81) with Paraquat (PS I electron diversion). There is no significant difference between Flumioxazin (PPO enzyme) and the control group. Chlorimuron (ALS enzyme) does not exhibit significant differences with most other treatments, except for the control and Atrazine (PS II inhibition). Similarly, Glyphosate (EPSPS synthase) and Glufosinate (Glutamine synthetase) do not show significant differences with most other treatments. Indaziflam (Cellulose synthesis) also cannot distinguish differences with the Amino acid synthesis inhibition MOA group herbicides (Chlorimuron, Glyphosate, and Glufosinate).

**Figure 5:**
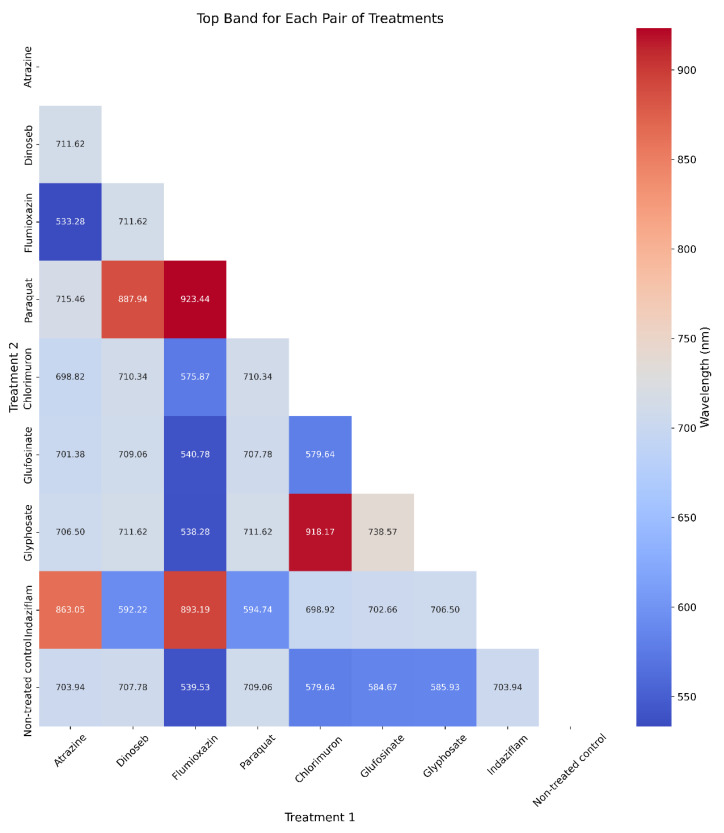
Heatmap of top band wavelengths for each treatment pair. Color intensity indicates the wavelength (in nm) of the top band, ranging from lower (cool colors) to higher (warm colors) wavelengths.

The band selection analysis for herbicide classification reveals unique patterns across various treatments. As shown in Figure 5, the featured band covered a wide range from 533.28 nm (Atrazine—PS II inhibition vs. Flumioxazin—PPO enzyme) to 923.44 nm (Flumioxazin—PPO enzyme vs. Paraquat—PS I electron diversion). In the differentiation between the treated and control groups, the feature bands of Glufosinate, Glyphosate, and Chlorimuron within the amino acid synthesis inhibition group are notably similar, ranging from 579.64 to 585.93 nm. Likewise, the Atrazine and Dinoseb feature bands in the photosynthesis inhibition group exhibit a close resemblance. However, the feature bands of Flumioxazin and Paraquat in the cell membrane disrupter group are distinctively located in the visible light region and infrared region, respectively.

### 3.2. Machine Learning Method Comparison Preliminary Result

**Figure 6:**
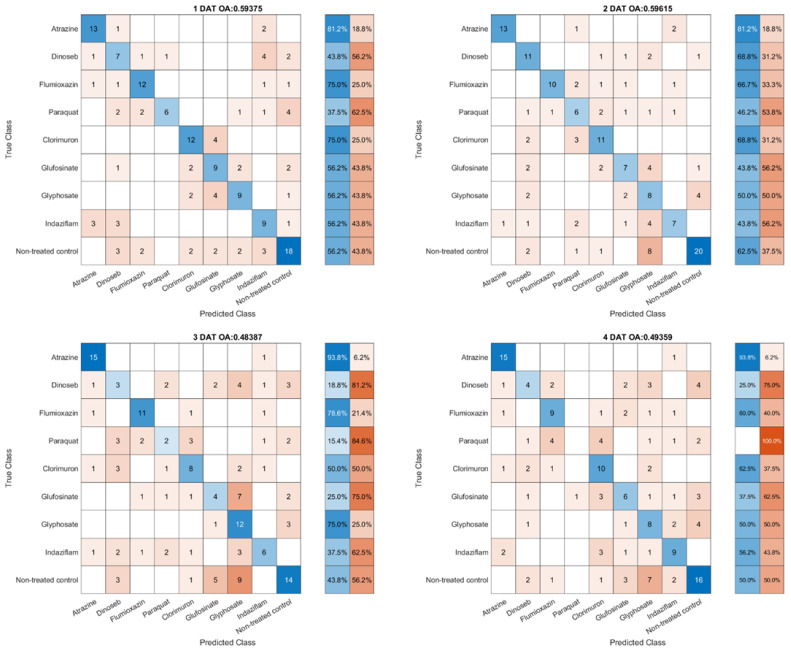
Leave-one-out cross-validation results from the PLSDA model with data collected from 1–4 DAT.

**Figure 7:**
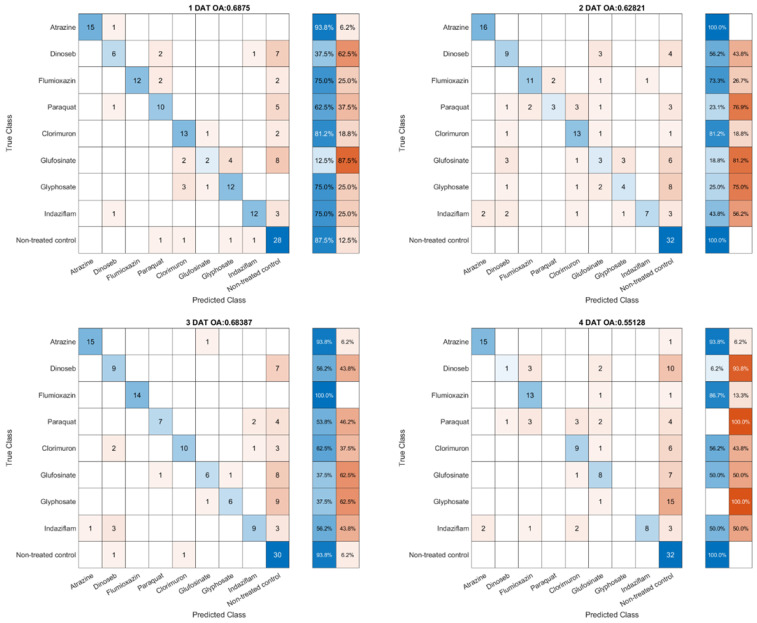
Leave-one-out cross-validation results from the SVM model with data collected from 1–4 DAT.

In the present study, a comparison was made between modeling methods, SVM and PLS-DA, for the classification of herbicides based on their SOA. This preliminary exploration serves as a foundational step in understanding the comparative efficacy and potential applicability of the chosen analytical approaches. The first-round data, consisting of hyperspectral images collected from 1–4 DAT, was used to train the models. The leave-one-out cross-validation results are presented in Figure 6 and Figure 7. The classification results are displayed in the form of confusion matrixes. The title of the matrix includes the Overall Accuracy (OA) and the corresponding DAT. The rightmost blue column in the matrix denotes the accuracy associated with each individual treatment, while the adjacent orange column indicates the respective error rates. The number of samples varies slightly with changes in DAT because a small subset of the samples experienced extensive damage and were not successfully captured by the imaging system. Additionally, some of these heavily damaged samples exhibited recovery in subsequent DAT evaluations.

In a comparative analysis, the results of the PLS-DA (Partial Least Squares Discriminant Analysis) model were found to be slightly inferior to those of the SVM (Support Vector Machine) model in terms of classification accuracy. Both models exhibited a decline in accuracy on 4 DAT, with the decrease predominantly influenced by the low accuracy rates associated with the herbicides dinoseb and paraquat. For the PLS-DA model, the classification accuracy for the control group was only around 50%, and misclassification occurred across various categories. In contrast, the SVM model consistently maintained an accuracy rate exceeding 90% for the control group, with most misclassifications involving the categorization of herbicides as control group members. However, on 4 DAT, 15 out of 16 Glyphosate were misidentified as healthy plants. In addition, herbicides achieved the highest classification accuracy on different days. For example, Atrazine achieved a classification rate of 93.8% at 4 DAT in the PLS-DA modeling result, and Glyphosate achieved the highest classification rate at 3 DAT. In this preliminary round of experimentation, a substantial number of misclassifications were observed for dinoseb, paraquat, and Glyphosate two days post-treatment. 

### 3.3. Classification Result Trained by Combined Round Data Set

**Figure 8:**
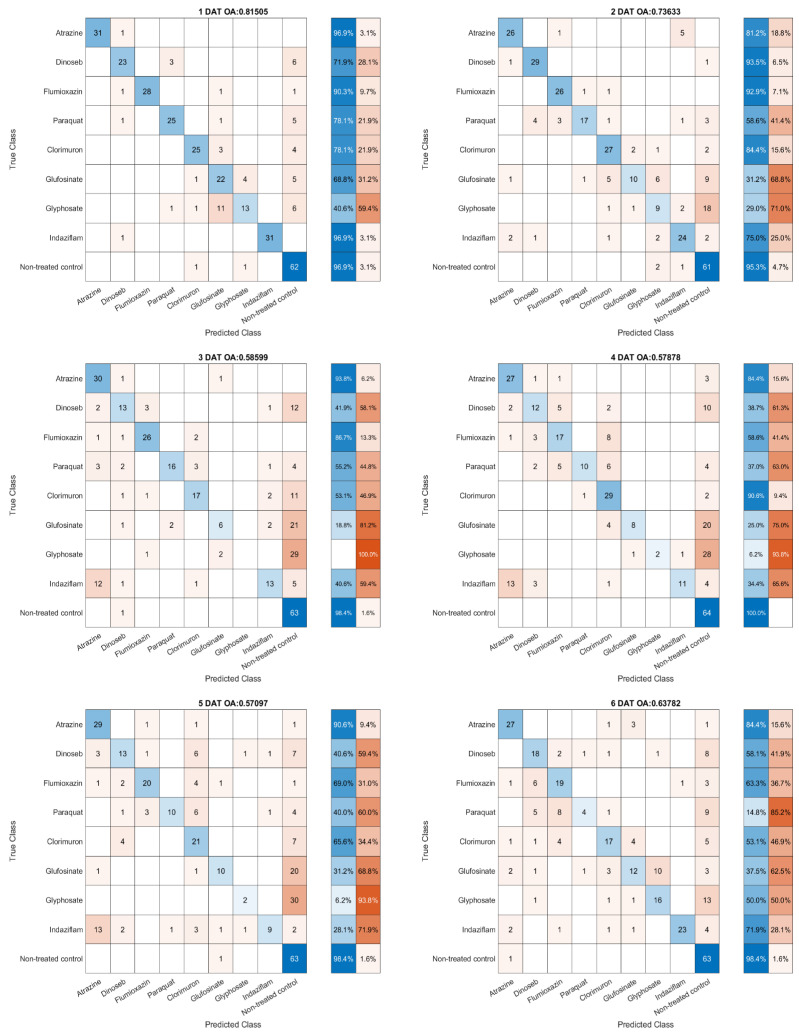

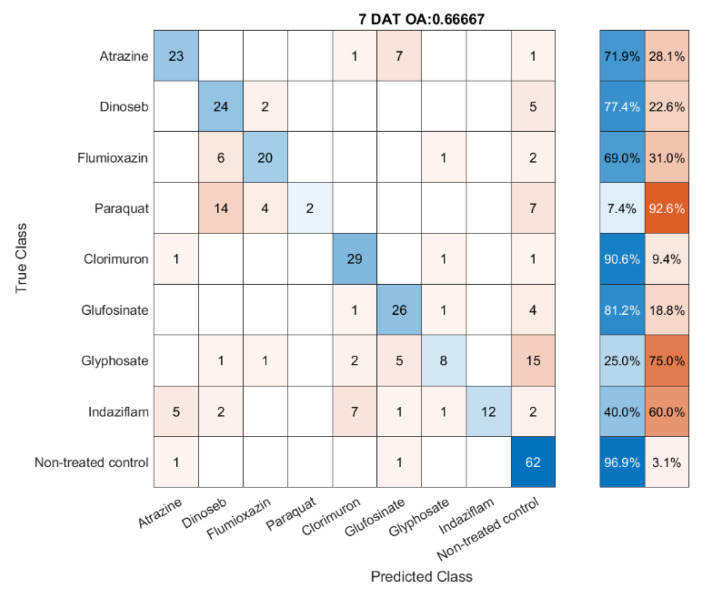
1–7 DAT herbicide SOA classification result trained by combined round data set.

The results of the combined round data set are shown in Figure 8.

The classification results of the SVM model trained with the combined round dataset showed the highest overall accuracy at 1 DAT. Specifically, the signal for Glyphosate (EPSPS synthase) was found to be difficult to detect prior to 6 DAT, while in contrast, the signal for paraquat diminished as the days progressed. However, with the introduction of additional data, there was a substantial improvement in the model’s classification results. Another notable finding was that at 1 DAT, the misclassification of Glyphosate (EPSPS synthase) was primarily concentrated in confusion with Glufosinate (Glutamine synthase). 

As shown in Table 4, herbicide belongs to different MOA groups that achieved the highest classification accuracy using models trained by different days’ data. Each herbicide’s corresponding MOA group, along with the specific day on which the best classification result was achieved, is depicted in the table below. This representation provides a concise overview of the classification performance, highlighting the temporal dynamics and the relationship between the herbicides’ SOA and their detectability.

Photosynthesis inhibitors such as Atrazine (PS II inhibition)and Dinoseb (Uncoupler) showed robust early detection, while Cell Membrane Disrupters like Flumioxazin (PPO enzyme) and Paraquat (PS I electron diversion) varied in signal strength. Amino Acid Synthesis Inhibitors presented mixed results, with Glyphosate’s (EPSPS synthase) detection notably lower and delayed. Indaziflam (Cellulose synthesis) demonstrated strong classification with 96.9% accuracy on 1 DAT.

### 3.4. Day-to-Day Validation Result

**Figure 9:**
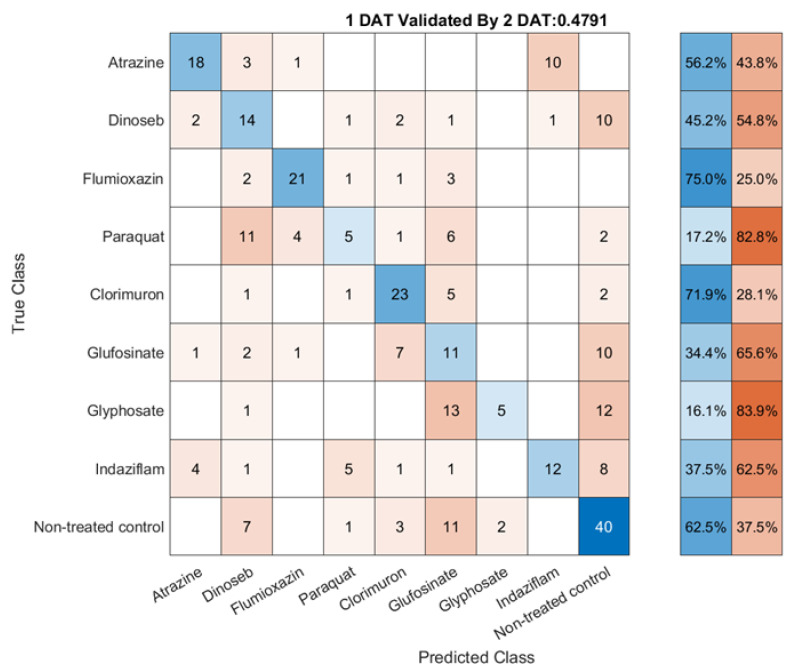
Day-to-day validation results.

Day-to-day validation was applied on 1 DAT model to avoid overfitting and test the stability of SVM models. The HSI data collected 2 DAT with rounds 1 and 2 combined and used as the validation dataset. The classification result confusion matrix is shown in Figure 9. 

In the validation results, it was observed that the accuracy of the SVM model varied for different herbicides, with the highest accuracy being shown by Chlorimuron (ALS enzyme) and Flumioxazin (PPO enzyme) at 71.9% and 75%, respectively. The UTC control group was found to have an accuracy of 62.5%. However, some herbicides, such as Paraquat (PS I electron diversion) and Glyphosate (EPSPS synthase), were found to have relatively low accuracy levels at 17.2% and 16.1%, respectively.

Despite the variation in accuracy, the overall performance of the model was found to be encouraging, with an overall accuracy of 47.9% on the first day after treatment (DAT) on a dataset with nine classes and 320 replicates in two rounds. 

## 4. Discussion

### 4.1. Herbicide Site of Action Distinction through Average NDVI Analysis

Reflecting both the complexity and specificity of herbicide impacts on vegetation, the analysis reveals various patterns across treatments. Atrazine’s significant differences with several treatments highlight its distinct effect on NDVI, a one-dimensional measurement that mainly captures the signal of chlorophyll activity. Thelen (2004), who employed NDVI to identify herbicide injury in soybeans, also highlighted the challenges encountered due to temporal and spatial variability in the crop’s response to the herbicide [28]. Conversely, the lack of significant differences for herbicides like Chlorimuron (ALS enzyme), Glyphosate (Glutamine synthase), and Glufosinate (EPSPS synthase) indicates a similar pattern of NDVI response across these comparisons. The inability of Indaziflam (Cellulose synthesis) to distinguish differences within the Amino acid synthesis inhibition SOA group may suggest a common MOA or similar impact on NDVI. Significant differences were observed between the Glyphosate-treated group and the control group, a finding that aligns with Ting Zhang’s study on corn plants. Glyphosate acts as a competitive inhibitor of the enzyme EPSP synthase, serving as a transition state analog. It exhibits stronger binding affinity to the EPSPS–S3P complex compared to phosphoenolpyruvate (PEP), thereby inhibiting the shikimate pathway. This disruption in enzymatic activity effectively halts the pathway, leading to a deficiency in essential aromatic amino acids [29]. The lack of aromatic amino acids leads to stress on the grass and is captured by the reflectance of the spectrum. 

In this study, as a task of multiclass classification, relying solely on average NDVI may not leverage the whole spectrum of information. Overall, these results contribute to the broader context of phenotyping and herbicide treatment classification, emphasizing the need for more advanced methods like machine learning to be developed. This nuanced approach could further the understanding of herbicide effects and facilitate more targeted and effective herbicide management.

### 4.2. Various Featured Bands Selected by Random Forest Method

According to the results obtained through the Random Forest selection method, the complexity of distinguishing herbicide behavioral patterns is vividly demonstrated. No single wavelength can differentiate all herbicides, as the characteristic wavelengths span from the green part of the visible light spectrum to the near-infrared region. This study identifies the key spectral bands for differentiating herbicides as falling within the green and yellow bands (533–579 nm), the red edge region (around 700 nm), and the near-infrared region. These findings are consistent with the results from Chu’s study on the classification of three different herbicides [16]. 

A key finding is that in the differentiation between the treated and control groups, the commonality in the mechanism of action among herbicides within the Amino Acid Synthesis Inhibition and Photosynthesis Inhibition groups is reflected in the spectral information. The distinct locations of the feature bands of Flumioxazin (PPO enzyme) in the NIR region and Paraquat (PS I electron diversion) within the Cell Membrane Disrupter group are intriguing. Earlier studies have indicated that spectral reflectance in the near-infrared (NIR) region is associated with cellular structure [30]. Furthermore, spectral bands in the green range can distinguish certain SOA. These include EPSP synthase, Glutamine synthase, and ALS enzyme. Previous research has shown that these green spectral features are reliable for measuring carotenoid content in green leaves and plants [31]. Regarding the red edge position, the inflection point on the curve between red absorption and near-infrared reflectance is commonly used as a correlate for chlorophyll content [32]. This may elucidate why Atrazine (PS II inhibition) and Dinoseb (Uncoupler) can be distinguished from other SOAs. Both belong to the Photosynthesis Inhibition Mode of Action group. Changes in chlorophyll content directly affect the photosynthesis process, making these herbicides distinct in their effects. Further experimentation is required to validate these findings, as this study represents the inaugural effort to utilize Hyperspectral Imaging (HSI) for classifying multiple SOA of herbicides. The underlying mechanisms warrant further exploration.

### 4.3. Understanding the SOA of Herbicides through the Integration of an SVM Models Trained on Combined-Round Datasets

The SVM models showed better performance compared to PLS-DA. This could be due to the nonlinearity that SVM has compared to the linear PLS-DA model. The spectral response (the input of the model) and the SOA of the herbicide (output of the model) are not linearly related.

The highest performance on 1 DAT can be attributed to several factors. Firstly, paraquat, dinoseb, and flumioxazin used in the study may have a rapid SOA, leading to rapid tissue death that makes it easier for the model to classify the herbicides’ effects [33,34]. Secondly, as the experiment progresses, the plants may develop stress symptoms that overlap between different herbicides or due to the recovery capacity of the plants [35]. These overlapping stress symptoms might make it more challenging for the model to differentiate between the herbicides’ effects in the later days of the experiment. 

Herbicides from different SOA attained the highest classification accuracy when models were trained using data from various days. This suggests that the optimal time for accurate classification may vary depending on the specific SOA of the herbicide in question. In other words, the day the data is collected for model training could impact the model’s ability to accurately classify herbicides based on their SOA. The possible reason is that Glufosinate is a little slower in inducing symptomology as products of inhibition must build up in the plant first [36]. Glyphosate and chlorimuron are also quite slow due to their amino acid starvation mechanism [37,38,39].

One challenge encountered in the study was that most misclassified replicates were incorrectly assigned to the untreated control group. At the given dosage of Glyphosate (15.6 g/ha), herbicide injury was not severe enough to be detected by the average spectrum. As previously outlined in the introduction, Ting Zhang (2021) employed HSI (Hyperspectral Imaging System) technology to detect glyphosate-induced damage signals in corn plants. Machine learning models were successful in capturing these signals as early as two weeks after treatment (2 WAT) [15]. Although the spectral responses to glyphosate may differ between corn and Barnyard grass, some common conclusions can be drawn from the results of these two studies. Specifically, the spectral signals generated by glyphosate-induced plant damage are often captured by machine-learning models only after an extended period. For subsequent research on the MOA, researchers should focus on collecting data between 1–2 weeks after treatment (1–2 WAT). 

### 4.4. Discussion on Day-to-Day Validation

One possible reason for the drop in accuracy for Paraquat (PS I electron diversion) and Dinoseb (Uncoupler) is that they have a quick effect, causing rapid death of the plant tissue within a few hours after spray. As a result, the spectral response of the treated tissue may be different on the first day after treatment (1DAT) compared to subsequent days. The capacity of recovery of the plant could potentially cause differences in the spectral features used by the machine learning models for these herbicides, resulting in lower accuracy on 2 DAT data.

In contrast, herbicides such as Chlorimuron (ALS enzyme) have slower effects on the plant tissue [17], allowing for a more gradual response and more consistent spectral features over multiple days. This could contribute to the higher accuracy observed for these herbicides.

This important information about validation results for herbicide SOA research suggests that there are varieties of SOA that have different development speeds. This leads to changes in hyperspectral data spectral information and stability sensitive to the time after treatments are applied. To build a stable model system that can classify multiple kinds of SOA, at least two rounds of data should be collected. If herbicides with different active speeds were conducted in the experiment, single-day data collection would not be enough since the spectrum will show herbicide damage signals on different days. Furthermore, the observed decline in accuracy emphasizes the limitations of solely relying on the average spectrum to construct models. This is due to the fact that many stress signals induced by herbicide damage do not uniformly result in color changes across the entire plant. For instance, low-dose application of dicamba may cause leaf deformation, which cannot be detected by the average spectrum alone [40]. By collecting high-resolution hyperspectral images of barnyard grass using tools like LeafSpec [41], it may be possible to capture these spatial signals and improve the accuracy of herbicide SOA detection.

## 5. Conclusions

This study presents a groundbreaking approach to classifying herbicide SOA using hyperspectral imaging and machine learning algorithms. Conducted in a fully automated, high-throughput imaging system, this study eliminates many microclimate variables and speeds up the herbicide development process. The study’s key innovation lies in its use of eight different herbicides across four SOAs, providing a basis for cross-MOA comparison. Advanced machine learning models, such as SVM, were employed, achieving over 80% classification accuracy as early as 1 day after treatment (DAT).

The main conclusions drawn from this study underscore the efficacy of hyperspectral imaging and machine learning in rapidly screening and precisely classifying a broad spectrum of herbicides. This capability is crucial for the isolation and detailed examination of prospective herbicide candidates. Moreover, the methodology adopted in this research advances beyond traditional assessment tools, such as NDVI, by leveraging complex spectral information to enhance the accuracy of SOA identification. Future work should integrate spatial information into hyperspectral data for even greater accuracy. Key considerations for future models include herbicide dosages, data collection time windows, and spatial and spectral features. The potential for significantly accelerating herbicide development is evident, especially if a well-curated hyperspectral database for herbicide SOA is developed. This could pave the way for a comprehensive decision-making model for faster herbicide development.

## Figures and Tables

**Table 1:** Parameters for the hyperspectral imaging sensor.

Parameters	Data
Camera model	MSV-500
Spectrograph	Specim V10E
Frame rate	30 FPS
Exposure time	6 ms
Spectral resolution	1.2 nm
Spectral range	399–1000 nm

**Table 2:** Sources of material for herbicide applied to experiment.

Common Name	Trade Name	Dose (g/ha)	Manufacturer
Atrazine	Aatrex	62.5	Syngenta—Greensboro, NC, USA
Chlorimuron	Classic	25	AMVAC Chemical Corporation -Newport Beach, CA, USA
Glufosinate	Liberty	62.5	BASF Corporation—Research Triangle Park, NC, USA
Glyphosate	Roundup PowerMax	15.6	Monsanto Company—St. Louis, MO, USA
Dinoseb	Dinoseb technical	125	Sigma-Aldrich—St. Louis, MO, USA
Flumioxazin	Valor SX	1.56	Valent U.S.A. Corporation—Walnut Creek, CA, USA
Indaziflam	Specticle Flo	62.5	Bayer Environmental Science—Research Triangle Park, NC, USA
Paraquat	Gramoxone 2SL	3.9	Syngenta—Greensboro, NC, USA

**Table 3:** Herbicide mode of action groups and site of action targets.

MOA Group	Herbicide	SOA Target
Photosynthesis inhibition	Atrazine	PS II inhibition
	Dinoseb	Uncoupler
Cell membrane disrupter	Flumioxazin	PPO enzyme
	Paraquat	PS I electron diversion
Amino acid synthesis inhibition	Glyphosate	EPSPS synthase
	Glufosinate	Glutamine synthase
	Chlorimuron	ALS enzyme
Cellulose biosynthesis inhibitor	Indaziflam	Cellulose synthesis

**Table 4:** Highest classification accuracy of herbicides by site of action.

Site of Action	Herbicide	Highest Accuracy	
PS II inhibition	Atrazine	1 DAT	96.9%
Uncoupler	Dinoseb	2 DAT	93.5%
PPO enzyme	Flumioxazin	2 DAT	92.9%
PS I electron diversion	Paraquat	1 DAT	78.1%
EPSPS synthase	Glyphosate	6 DAT	50%
Glutamine synthase	Glufosinate	7 DAT	81.2%
ALS enzyme	Chlorimuron	4/7 DAT	90.6%
Cellulose synthesis	Indaziflam	1 DAT	96.9%

## Data Availability

The data presented in this study are available on request from the corresponding author. The data are not publicly available due to confidentiality.

## References

[1] Duke S.O., Stidham M.A., Dayan F.E. (2019). A Novel Genomic Approach to Herbicide and Herbicide Mode of Action Discovery. Pest Manag. Sci..

[2] Dayan F.E. (2019). Current Status and Future Prospects in Herbicide Discovery. Plants.

[3] Godfray H.C.J., Beddington J.R., Crute I.R., Haddad L., Lawrence D., Muir J.F., Pretty J., Robinson S., Thomas S.M., Toulmin C. (2010). Food Security: The Challenge of Feeding 9 Billion People. Science.

[4] Mascarelli A. (2013). Growing Up With Pesticides. Science.

[5] Sparks T.C., Lorsbach B.A. (2017). Perspectives on the Agrochemical Industry and Agrochemical Discovery. Pest Manag. Sci..

[6] Duke S.O. (2012). Why Have No New Herbicide Modes of Action Appeared in Recent Years?. Pest Manag. Sci..

[7] Duke S.O., Owens D.K., Dayan F.E. (2014). The Growing Need for Biochemical Bioherbicides. Biopesticides: State of the Art and Future Opportunities.

[8] He B., Hu Y., Wang W., Yan W., Ye Y. (2022). The Progress towards Novel Herbicide Modes of Action and Targeted Herbicide Development. Agronomy.

[9] Chiddarwar R.K., Rohrer S.G., Wolf A., Tresch S., Wollenhaupt S., Bender A. (2017). In Silico Target Prediction for Elucidating the Mode of Action of Herbicides Including Prospective Validation. J. Mol. Graph. Model..

[10] Lamberth C., Jeanmart S., Luksch T., Plant A. (2013). Current Challenges and Trends in the Discovery of Agrochemicals. Science.

[11] Manning L. Bayer Is Betting on Chemical Ag with New Herbicide Slated for 2030. What about Biologics?. https://agfundernews.com/bayer-is-betting-on-chemical-ag-with-new-herbicide-slated-for-2030-what-about-biologics.

[12] Ofosu R., Agyemang E.D., Márton A., Pásztor G., Taller J., Kazinczi G. (2023). Herbicide Resistance: Managing Weeds in a Changing World. Agronomy.

[13] Brodie G., Jabran K., Chauhan B.S. (2018). Chapter 3—The Use of Physics in Weed Control. Non-Chemical Weed Control.

[14] Herbicide Resistant Weeds by Herbicide Site of Action Summary Table. https://www.weedscience.org/Pages/SOASummary.aspx.

[15] Zhang T., Huang Y., Reddy K.N., Yang P., Zhao X., Zhang J. (2021). Using Machine Learning and Hyperspectral Images to Assess Damages to Corn Plant Caused by Glyphosate and to Evaluate Recoverability. Agronomy.

[16] Chu H., Zhang C., Wang M., Gouda M., Wei X., He Y., Liu Y. (2022). Hyperspectral Imaging with Shallow Convolutional Neural Networks (SCNN) Predicts the Early Herbicide Stress in Wheat Cultivars. J. Hazard. Mater..

[17] Niu Z. (2022). Early Detection of Dicamba and 2,4-D Herbicide Injuries on Soybean with LeafSpec, an Accurate Handheld Hyperspectral Leaf Scanner. Master’s Thesis.

[18] Ma D., Carpenter N., Amatya S., Maki H., Wang L., Zhang L., Neeno S., Tuinstra M.R., Jin J. (2019). Removal of Greenhouse Microclimate Heterogeneity with Conveyor System for Indoor Phenotyping. Comput. Electron. Agric..

[19] Shaikh M.S., Jaferzadeh K., Thörnberg B., Casselgren J. (2021). Calibration of a Hyper-Spectral Imaging System Using a Low-Cost Reference. Sensors.

[20] Zhang L., Maki H., Ma D., Sánchez-Gallego J.A., Mickelbart M.V., Wang L., Rehman T.U., Jin J. (2019). Optimized Angles of the Swing Hyperspectral Imaging System for Single Corn Plant. Comput. Electron. Agric..

[21] Savitzky A., Golay M.J.E. (1964). Smoothing and Differentiation of Data by Simplified Least Squares Procedures. Anal. Chem..

[22] Cabrera-Bosquet L., Molero G., Stellacci A., Bort J., Nogués S., Araus J. (2011). NDVI as a Potential Tool for Predicting Biomass, Plant Nitrogen Content and Growth in Wheat Genotypes Subjected to Different Water and Nitrogen Conditions. Cereal Res. Commun..

[23] Fei H., Fan Z., Wang C., Zhang N., Wang T., Chen R., Bai T. (2022). Cotton Classification Method at the County Scale Based on Multi-Features and Random Forest Feature Selection Algorithm and Classifier. Remote Sens..

[24] Advanced Preprocessing: Sample Normalization—Eigenvector Research Documentation Wiki. https://wiki.eigenvector.com/index.php?title=Advanced_Preprocessing%3A_Sample_Normalization.

[25] Rehman T.U., Zhang L., Wang L., Ma D., Maki H., Sánchez-Gallego J.A., Mickelbart M.V., Jin J. (2020). Automated Leaf Movement Tracking in Time-Lapse Imaging for Plant Phenotyping. Comput. Electron. Agric..

[26] Guo Y., Yin X., Zhao X., Yang D., Bai Y. (2019). Hyperspectral Image Classification with SVM and Guided Filter. EURASIP J. Wirel. Commun. Netw..

[27] Wang Y., Duan H. (2018). Classification of Hyperspectral Images by SVM Using a Composite Kernel by Employing Spectral, Spatial and Hierarchical Structure Information. Remote Sens..

[28] Thelen K.D., Kravchenko A.N., Lee C.D. (2004). Use of Optical Remote Sensing for Detecting Herbicide Injury in Soybean. Weed Technol..

[29] Schönbrunn E., Eschenburg S., Shuttleworth W.A., Schloss J.V., Amrhein N., Evans J.N.S., Kabsch W. (2001). Interaction of the Herbicide Glyphosate with Its Target Enzyme 5-Enolpyruvylshikimate 3-Phosphate Synthase in Atomic Detail. Proc. Natl. Acad. Sci. USA.

[30] Wei C., Huang J., Wang X., Blackburn G.A., Zhang Y., Wang S., Mansaray L.R. (2017). Hyperspectral Characterization of Freezing Injury and Its Biochemical Impacts in Oilseed Rape Leaves. Remote Sens. Environ..

[31] Zur Y., Gitelson A., Chivkunova O., Merzlyak M. The Spectral Contribution of Carotenoids to Light Absorption and Reflectance in Green Leaves. Proceedings of the 2nd International Conference Geospatial Information in Agriculture and Forestry, Lake Buena Vista.

[32] Horler D.N.H., Dockray M., Barber J. (1983). The Red Edge of Plant Leaf Reflectance. Int. J. Remote Sens..

[33] Jordan T.N., Warren G.F. (1975). Effects of Prometryn and Dinoseb Combinations in an Undiluted Oil Carrier. Weed Sci..

[34] Henry W.B., Shaw D.R., Reddy K.R., Bruce L.M., Tamhankar H.D. (2004). Remote Sensing to Detect Herbicide Drift on Crops. Weed Technol..

[35] Suarez L.A., Apan A., Werth J. (2017). Detection of Phenoxy Herbicide Dosage in Cotton Crops through the Analysis of Hyperspectral Data. Int. J. Remote Sens..

[36] Takano H.K., Beffa R., Preston C., Westra P., Dayan F.E. (2020). A Novel Insight into the Mode of Action of Glufosinate: How Reactive Oxygen Species Are Formed. Photosynth. Res..

[37] Chahal G.S., Johnson W.G. (2012). Influence of Glyphosate or Glufosinate Combinations with Growth Regulator Herbicides and Other Agrochemicals in Controlling Glyphosate-Resistant Weeds. Weed Technol..

[38] Claus J.S. (1987). Chlorimuron-Ethyl (Classic)^®^: A New Broadleaf Postemergence Herbicide in Soybean. Weed Technol..

[39] Reddy K.N., Bryson C.T., Burke I.C. (2007). Ragweed Parthenium (*Parthenium hysterophorus*) Control with Preemergence and Postemergence Herbicides. Weed Technol..

[40] Robinson A.P., Simpson D.M., Johnson W.G. (2013). Response of Glyphosate-Tolerant Soybean Yield Components to Dicamba Exposure. Weed Sci..

[41] Wang L., Jin J., Song Z., Wang J., Zhang L., Rehman T.U., Ma D., Carpenter N.R., Tuinstra M.R. (2020). LeafSpec: An Accurate and Portable Hyperspectral Corn Leaf Imager. Comput. Electron. Agric..

